# Independently Outgrowing Neurons and Geometry-Based Synapse Formation Produce Networks with Realistic Synaptic Connectivity

**DOI:** 10.1371/journal.pone.0085858

**Published:** 2014-01-16

**Authors:** Arjen van Ooyen, Andrew Carnell, Sander de Ridder, Bernadetta Tarigan, Huibert D. Mansvelder, Fetsje Bijma, Mathisca de Gunst, Jaap van Pelt

**Affiliations:** 1 Department of Integrative Neurophysiology, Center for Neurogenomics and Cognitive Research, VU University Amsterdam, Amsterdam, The Netherlands; 2 Department of Mathematics, VU University Amsterdam, Amsterdam, The Netherlands; Newcastle University, United Kingdom

## Abstract

Neuronal signal integration and information processing in cortical networks critically depend on the organization of synaptic connectivity. During development, neurons can form synaptic connections when their axonal and dendritic arborizations come within close proximity of each other. Although many signaling cues are thought to be involved in guiding neuronal extensions, the extent to which accidental appositions between axons and dendrites can already account for synaptic connectivity remains unclear. To investigate this, we generated a local network of cortical L2/3 neurons that grew out independently of each other and that were not guided by any extracellular cues. Synapses were formed when axonal and dendritic branches came by chance within a threshold distance of each other. Despite the absence of guidance cues, we found that the emerging synaptic connectivity showed a good agreement with available experimental data on spatial locations of synapses on dendrites and axons, number of synapses by which neurons are connected, connection probability between neurons, distance between connected neurons, and pattern of synaptic connectivity. The connectivity pattern had a small-world topology but was not scale free. Together, our results suggest that baseline synaptic connectivity in local cortical circuits may largely result from accidentally overlapping axonal and dendritic branches of independently outgrowing neurons.

## Introduction

Electrical activity dynamics underlying cognitive function strongly depends on the organization of synaptic connectivity. The connectivity structure in cortical circuits determines how information is transmitted and what spatiotemporal patterns of network activity can arise [1]–[4]. At the neuron level, the spatial distribution of synapses on dendrites critically influences input integration and neuronal signal processing [5], [6]. Altered patterns of synaptic connectivity have been implicated in brain disorders such as autism [7], schizophrenia [8]–[10] and Alzheimer's disease [11].

During development, neurons can establish synaptic connections when their axonal and dendritic branches come into close proximity of each other [12]. A large variety of signaling mechanisms, such as extracellular chemical attraction and repulsion, has been shown to play a role in guiding and positioning axonal branches, shaping dendritic morphology and creating specific patterns of synaptic connectivity [13]–[17].

In addition to these chemospecific mechanisms, which enable neurons to interact with each other and selectively steer their neuronal arbors prior to synapse formation, the geometry of neuronal arborizations by itself is also expected to be an important determinant of synaptic connectivity [18]. Synapse formation requires close spatial apposition of axonal and dendritic branches, and the locations where this occurs depend on the metrical and topological properties of the axonal and dendritic branching patterns. However, the extent to which accidental appositions resulting from overlapping axonal and dendritic morphologies of independently outgrowing neurons can account for synapse distributions and connectivity patterns remains unclear [18], [19]. Previous studies examining the relationship between neuronal morphology and synaptic connectivity in local cortical circuits focused only on particular aspects of connectivity [19]–[22] or employed highly abstract neuronal morphologies [23].

To explore what synaptic connectivity patterns can arise from neuronal morphology alone, we generated a local 3D network of independently outgrowing and morphologically realistic rat cortical L2/3 neurons among which synapses were formed solely on the basis of proximity between axonal and dendritic branches. We subsequently analyzed a wide range of features of the emerging patterns of synaptic connectivity and spatial distributions of synapses. To generate neurons, we used our simulation framework NETMORPH, introduced in [24]. Here, we applied NETMORPH for the first time in a full study, analyzing many features of synaptic connectivity. Our model results show that realistic neuronal morphologies, simple geometry-based synapse formation rules and independently developing neurons are capable of producing networks with realistic synapse distributions, connectivity patterns and small-world topology.

## Methods

To build a neuronal network, we first collected rat cortical L2/3 pyramidal neurons from the NeuroMorpho.org database (http://neuromorpho.org) [25] and analyzed their morphological shape characteristics. The choice of L2/3 pyramidal neurons was motivated by the availability of experimental data on morphological reconstructions and synaptic connectivity. The morphological shape characteristics were then used to obtain parameter values for the neurite growth model in NETMORPH in order to generate model neurons that were as similar as possible to the empirical neurons. Next, we generated a network of independently outgrowing neurons in NETMORPH using these optimized growth parameter values. We subsequently positioned synapses on locations where axonal and dendritic branches came within a threshold distance of each other, and analyzed the emerging synapse distributions and connectivity patterns. Thus, importantly, the parameter values of the neurite outgrowth model were optimized only for generating realistic neuronal morphologies and not for producing particular connectivity patterns. From a functional point of view, the synapses formed in the model network should be viewed as candidate or potential synapses, but for simplicity we will refer to them as synapses.

In the following sections, we briefly describe neuronal morphogenesis and synapse formation in NETMORPH, the shape characteristics used to quantify the morphology of empirical and model-generated neurons, the optimization procedure for finding parameter values for the neurite outgrowth model, the set of measures used to quantify the synapse distributions and connectivity patterns in the network, and an overview of the workflow of our study.

### NETMORPH program

NETMORPH is a modular simulation tool for generating networks with realistic neuron morphologies [24]. NETMORPH simulates the development of neuron morphology by using stochastic growth rules for the behavior of individual growth cones (the structures at the tip of outgrowing neurites that mediate neurite elongation and branching). Neurons are positioned in 3D space and grow out independently of each other. Synapses between neurons are formed when crossing axonal and dendritic segments come sufficiently close to each other [26]. NETMORPH is available from http://www.neurodynamics.nl.

The neurite outgrowth model implemented in NETMORPH is based on the stochastic rules for neurite branching and elongation that have been formulated by Van Pelt et al. [27], [28] and that have been shown to generate realistic neuronal morphologies [29], [30]. The model is a phenomenological model based on a stochastic description of growth actions, meaning that it implicitly incorporates both intrinsic mechanisms and external factors that affect the development of neuronal morphology. In brief, each growth cone has at each time step a probability to elongate the trailing neurite, to branch and produce two daughter growth cones, and to turn and change the direction of neurite outgrowth [31], [32]. Branching and elongation are modelled as independent processes, so they can be validated separately. The neurite outgrowth model was used for generating both dendritic and axonal arborizations, but with different parameter values.

### Neurite branching

**Figure 1:**
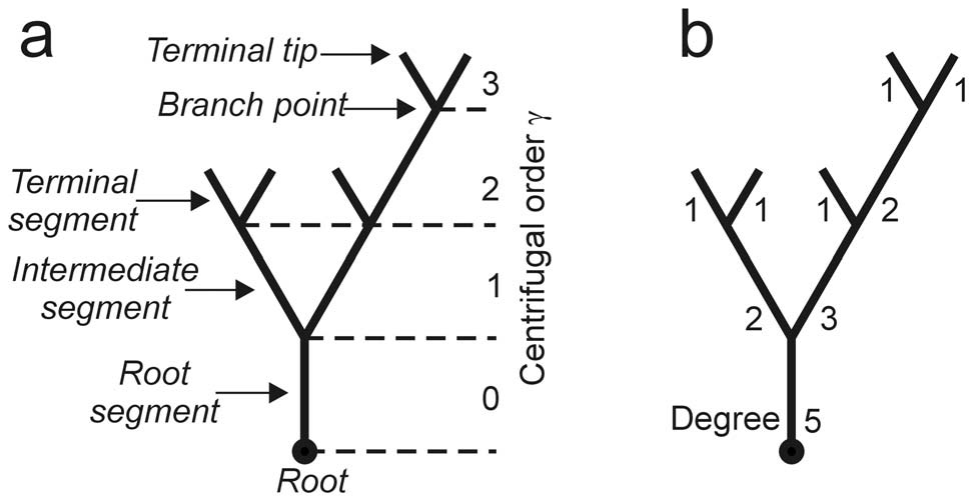
Schematic neuritic trees illustrating tree terminology. **a**, The different segments and nodes that can be distinguished and the labeling of segments based on centrifugal order. The centrifugal order of a segment is the number of branch points along the path from the root to the terminal tip of the segment. Terminal tip is equivalent to growth cone. **b**, Labeling of segments based on degree. The degree of a segment is the number of terminal tips in the (sub) tree carried by the segment.

Each terminal segment *j* (see Fig. 1 for terminology) branches in a discrete time step 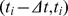 with probability 
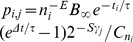
 (for the derivation of this equation, see [24]). The term 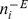 makes the branching probability dependent on the momentary number 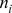 of terminal segments in the tree, with parameter *E* (called competition parameter) modulating the strength of this dependency. The term 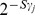 makes the branching probability dependent on the centrifugal order *γ* of the terminal segment, with parameter *S* modulating the strength of this dependency. The coefficient 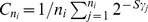 normalizes at each time point the order dependency of all tips. The term 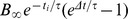 is the time-dependent baseline branching rate, representing all factors that influence branching but that are not covered by the dependence on the total number of terminal segments in the tree, where *τ* is a time constant and 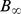
is the asymptotic expected number of branching events at a tip for *E* = 0. The 3D outgrowth directions of the daughter branches after a branching event were determined as described in [24]. The values of the parameters *B*_∞_, *τ*, *S* and *E* were optimized so as to obtain an optimal match with the morphology of empirical L2/3 pyramidal neurons (see Methods, Parameter optimization).

### Neurite elongation

Given that the rate of neurite elongation can vary considerably [33], also on the time scale of the chosen time step Δ*t* of 200 s, the new daughter growth cones that are produced by a branching event are assigned individual growth rates, which they maintain until they themselves experience a branching event. The elongation rates are obtained by random sampling from a Gaussian distribution, with mean and standard deviation eri-mn and eri-sd, respectively (NETMORPH parameters; eri stands for elongation rate initialization). During elongation, neurites can also change their direction (neurite turning), as described in [24]. The values of eri-mn and eri-sd (Table 1) were optimized to obtain an optimal match with the morphology of L2/3 pyramidal neurons (see Methods, Parameter optimization).

**Table 1:** Optimized values of the neurite outgrowth parameters in NETMORPH.

			Apical dendrite		
Growth parameter	Axon	Basal dendrites	Main stem	Tuft	Obliques
*B*_∞_	13.2	2.52	0.1	25	1.5
*E*	0.319	0.73	0	0.3	0.3
*S*	−0.205	0.5	0	1	1
*τ* (s)	1681541	259680	400000	400000	500000
eri-mn (µm/s)	0.000214	0.0000914	0.00102	0.000225	0.00004
eri-sd (µm/s)	0.000398	0.0000366	0.000026	0.000004	0.000001
Δ*t* (s)	200	200	200	200	200
days	18	18	18	18	18
trunk length-mn (µm)			80		
trunk length-sd (µm)			2		

### Synapse formation

**Figure 2:**
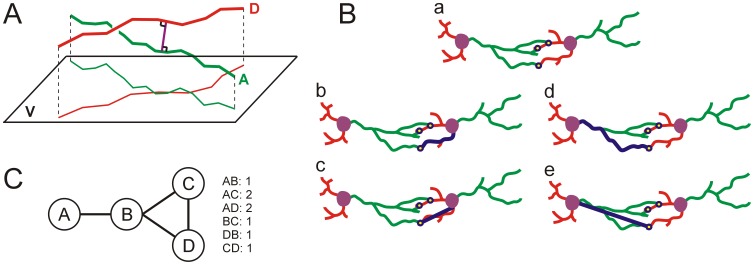
Synapse formation and illustration of measures characterizing synapse location and connectivity. **A**, Synapse formation in NETMORPH. The shortest distance between an axonal (A) and a dendritic (D) branch is defined as the orthogonal distance between a pair of crossing axonal and dendritic line pieces. If this shortest distance is smaller than a given threshold value, the orthogonal line (purple) marks the location of a synapse. **B**, Different ways to express synapse location. **a**, Two connected cells showing axons (green), dendrites (red), somata (purple) and synapses (yellow circles). **b**, Postsynaptic path distance (blue line). **c**, Postsynaptic Euclidean distance. **d**, Presynaptic path distance. **e**, Presynaptic Euclidean distance. **C**, Determining the mean shortest path length and clustering coefficient of an undirected graph consisting of four nodes and four edges. The numbers are the path lengths between the nodes. The mean shortest path length of the graph is the average of these numbers, 1.3333. The clustering coefficients of nodes A, B, C and D are 0, 2/6, 2/2, and 2/2, respectively. The clustering coefficient of the graph is the average of these numbers, 0.5833.

Synapse locations are defined as those places in the 3D meshwork of axonal and dendritic arborizations at which axons and dendrites come within a threshold distance of each other. Because the model-generated neurons are represented by piecewise-linear elements (lines or cylinders, with a length of a few microns, as determined by the NETMORPH parameters for neurite turning [24]), the proximity test needs to be performed on all pairs of axonal and dendritic line pieces. To be regarded as a synapse location, the current version of NETMORPH requires that the axonal and dendritic line pieces cross and that the orthogonal distance between them (taken from the centre lines of the axonal and dendritic cylinders) is smaller than a threshold value (Fig. 2A). Considering that in biological neurons the diameter of neurites is around 2 µm and the length of filopodia (providing an extended range for sensing other neurites) around 1 µm [34], we chose a threshold value of 4 µm by default. NETMORPH searches for synapse locations at the end of the growth process, when all neurons are completely formed. Alternatively, the search can be performed during outgrowth, but this yields exactly the same results because there are no interactions between the cells. At each synapse location found, a single synapse between axon and dendrite is established. The algorithm for finding synapses in the current version of NETMORPH was developed in [26].

### Morphological shape characteristics

Both topological and metrical measures (see Fig. 1) were used to characterize and quantify the neuritic tree morphology (from a model-generated or an empirical neuron).

The *degree* of a neuritic tree is equal to the number of its terminal tips. Since each segment in a tree can be regarded as the root segment of a subtree, each segment can be labeled by the degree of the subtree it carries. The segments can also be labeled by their (topological) distance from the root. The centrifugal order of a segment is the number of branch points along the path from the root to the distal end of the segment. The *mean centrifugal order* of a tree is the average centrifugal order of all the terminal and intermediate segments.

The *total length* of a tree is the sum of the lengths of all the segments in the tree. The *mean intermediate segment length* is the average length of all the intermediate segments, and the *mean terminal segment length* is the average length of all the terminal segments. The path length of a terminal tip is the total length of all the segments on the path from the root to the terminal tip. The *mean path length* is the average of all the path lengths from the root to the terminal tips.

### Parameter optimization

The morphological characteristics of the NETMORPH-generated neurons depend on six parameters (*E*, *S*, *B*_∞_, *τ*, eri-mn and eri-sd) parameterizing the neurite outgrowth model (see Methods, Neurite branching and Neurite elongation). We used a genetic algorithm to find parameter values producing morphological characteristics of the generated neurons that were as similar as possible to those of the L2/3 neurons obtained from the NeuroMorpho.org database [25]. The generated neurons were compared with empirical neurons with respect to six shape characteristics: two topological measures (degree and centrifugal order) and four metrical measures (total tree length, intermediate segment length, terminal segment length and mean path length). The optimization was done with regard to both the means and the standard deviations of the distributions of these six shape characteristics (thus in total 12 measures were considered).

The genetic algorithm initially created sets of outgrowth parameters with random values, subsequently simulated neuronal morphologies for each of these sets, and then iteratively improved the parameter sets. The optimization of axonal and dendritic trees was done separately. In addition, a distinction was made between basal and apical dendritic trees, with the apical dendrite further divided into main stem, oblique dendrites and apical tuft. A parameter set had a fitness value, indicating how well the shape characteristics of the trees generated by the parameter values matched the characteristics of the empirical trees. This fitness was defined as 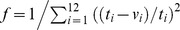, where *i* refers to a shape characteristic (mean or standard deviation), *t_i_* is the value of this characteristic in the empirical trees, and ν*_i_* is the corresponding value of that characteristic in the model-generated trees. Parameter sets were randomly selected according to their fitness value and then combined, subject to crossover and mutation, to form a new generation of parameter sets.

### General connectivity measures

A broad set of connectivity measures was used to characterize the emergent synapse distributions and network connectivity in the generated network.

Two neurons have a *connection* when they share at least one synaptic contact. The *connection strength* is the number of synapses from a presynaptic neuron onto the dendrites of a postsynaptic neuron. The *connection length* is the Euclidean distance between the somata of two connected neurons. The *connection probability* between two neurons is the probability that two randomly selected neurons in a network are connected, i.e., have at least one synaptic contact from the axon of one of the neurons onto the dendrite of the other neuron. The *connection probability versus Euclidean distance* is the probability that two randomly selected neurons in a network with their somata at a given Euclidean distance from each other are connected.

#### Synaptic distance to post- or presynaptic soma

The position of a synapse can be given as its distance to the postsynaptic soma or to the presynaptic soma (Fig. 2B). Both quantities can be expressed either in path distance or in Euclidean distance. Thus, there are four measures in total: the path distance of a synapse along the dendrite to the postsynaptic soma, the Euclidian distance of a synapse to the postsynaptic soma, the path distance of a synapse along the axon to the presynaptic soma, and the Euclidean distance of a synapse to the presynaptic soma.

### Graph theoretical measures

To characterize and quantify the emergent connectivity in the NETMORPH-generated network, we also used several measures from graph theory. In graph terminology, neurons are nodes and connections are edges. A distinction can be made between directed graphs, in which edges carry the direction of the axon-to-dendrite signal flow, and undirected graphs, in which edges do not carry such information.

#### In- and out-degree and degree

In a directed graph, the in-degree of a node is the number of nodes from which it receives an incoming connection (in our case, with at least one synapse), whereas the out-degree of a node is the number of nodes to which it projects an outgoing connection (in our case, with at least one synapse). The degree of a node (not to be confused with the degree of a tree; see Methods, Morphological shape characteristics) is the total number of nodes to which it is connected by incoming and outgoing connections. Highly connected nodes are known as hubs and are considered important for information processing [35].

#### Mean shortest path length

The shortest path from a node to another node is the path with the fewest edges. The mean shortest path length of a node (not to be confused with the mean path length of a tree; see Methods, Morphological shape characteristics) is the average of the node's shortest path lengths to all nodes in the network. The mean shortest path length of a graph is the average of all shortest path lengths between all node pairs [36] (Fig. 2C). Calculation of the mean shortest path length is straightforward in connected, undirected graphs. However, in directed or unconnected graphs, non-existing paths may occur. One approach to dealing with non-existing paths is to average only over existing paths in the calculation of the mean shortest path length [37]. Another approach is to analyze the unconnected sub-graphs separately. In the NETMORPH-generated network, we found that all neurons were connected, so our results were not affected by non-existing paths. The network was analyzed as an undirected graph.

#### Clustering coefficient

This describes to what extent a node's neighbors are interconnected. The clustering coefficient of a node in an undirected graph is calculated as 
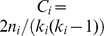
, where *n_i_* is the number of edges among the neighbors of node *i* (i.e., the nodes connected to node *i*) and *k_i_* is the number of neighbors of node *i* [36]. The clustering coefficient of the entire network is the average of the clustering coefficients of all the nodes (Fig. 2C).

#### Small-world topology

In small-world networks, most nodes can reach each other in only a few steps (small mean shortest path length) and the neighbors of a node have a high degree of interconnectivity (high clustering coefficient). Small-world networks are situated between random networks (small mean shortest path length, low clustering coefficient) and regular networks (large mean shortest path length, high clustering coefficient) [36]. Small-world topology supports both information segregation and rapid information transfer [38]–[41] and may improve memory recall [42]. The small-worldness of a network is calculated by determining the ratio *γ/λ*, where *γ* = *C*_original_/*C*_random_ and *λ* = *L*_original_/*L*_random_, with *C* denoting the clustering coefficient, *L* denoting the mean shortest path length, and ‘random’ denoting a randomized version (with the same number of nodes and edges) of the original network. In small-world networks, *λ* will be approximately one, *γ* greater than one, and *γ/λ* therefore also greater than one [36]. A randomized network is created by randomly redistributing the edges among the nodes [43]. Randomization may lead to non-existing paths and unconnected sub-graphs, which influence the calculation of the mean shortest path length. We therefore checked whether in the randomized versions of the NETMORPH-generated network all the nodes were still reachable. In all randomizations, it turned out that all nodes remained connected.

### Workflow

The workflow of our study consisted of the following consecutive steps:

#### Collecting experimental data and analyzing morphological shape characteristics

Experimental reconstructions of rat cortical L2/3 pyramidal neurons were obtained from the NeuroMorpho.org database [25], particularly from the data of Shepherd and Svoboda [44]. These reconstructions were subsequently analyzed for their morphological shape characteristics (see Methods, Morphological shape characteristics). Ten neurons from the database were used for analyzing dendritic trees (ten apical dendritic trees and 42 basal dendritic trees; note that a pyramidal neuron has several basal dendritic trees). For analyzing axons, four neurons from the database had sufficiently complete axonal trees (see also Discussion).

#### Optimizing neurite outgrowth parameters

The shape characteristics of these L2/3 pyramidal neurons were then used to optimize the neurite outgrowth parameters in NETMORPH by means of a genetic algorithm, separately for basal dendrites, apical dendrites and axons. The apical dendrite was further decomposed into its main stem, apical tuft and oblique dendrites, which were all treated separately in the parameter optimization. The axon was not further divided into subparts. Importantly, the parameter values of the neurite outgrowth model were optimized only for generating realistic neuronal morphologies, not for producing particular connectivity patterns.

#### Generating a network of model neurons

The optimized growth parameter values were then used to generate a network of model neurons in NETMORPH. The network represented a single cortical layer with 250 pyramidal neurons. (Simulations with more neurons were not feasible because of the heavy load on computer resources.) The neuronal cell bodies were randomly placed, with a minimum distance between the somata of 20 µm, in a 3D disc-shaped area with a height of 360 µm (the typical cortical thickness of L2/3 [45]) and a radius of 93 µm. This procedure yielded a density of about 25000 neurons per mm^3^, which is of the same order of magnitude as that reported for the rat visual cortex [20]. Soma diameters were drawn from a given normal distribution. The initial segment of an axon was oriented downwards, opposite to the direction of the initial segment of the apical dendrite. For each pyramidal cell, the number of basal dendrites was randomly drawn from a uniform distribution between 4 and 8. The total simulation time corresponded to a developmental period of about 18 days, the time in which L2/3 pyramidal cells reach their mature size in rat cortex [46]. The time step Δ*t* of the neurite outgrowth model was 200 s.

#### Locating synapses in the generated network

Synapses were positioned on the basis of the proximity between crossing axonal and dendritic segments [26], with a default distance criterion of 4 µm (see Methods, Synapse formation).

#### Analyzing synapse locations and network connectivity in the generated network

Finally, the synapse distributions and network connectivity in the generated network were analyzed, and compared with experimental data, with respect to the following features: distance from synapse to pre- and postsynaptic soma along axon and dendrite, respectively; Euclidean distance from synapse to pre- and postsynaptic soma; number of synapses by which neurons are connected; connection probability as a function of distance between somata; distance between somata of connected neurons; in- and out-degree of neurons; and small-world topology of synaptic connectivity.

## Results

To investigate whether the accidental overlap between axonal and dendritic branches can account for synapse distributions and connectivity patterns, we created a 3D network of independently outgrowing rat cortical L2/3 neurons using our simulation framework NETMORPH [24]. First, we show that NETMORPH produced realistic neuronal morphologies of L2/3 pyramidal neurons. Secondly, we describe the emerging synapse distributions and connectivity patterns in the NETMORPH-generated network and compare these to the available experimental data on synaptic connectivity.

### Neuronal morphology

Table 1 shows the parameter values that were found by the parameter optimization (see Methods, Parameter optimization) and that were used in the neurite outgrowth model of NETMORPH to generate the dendritic and axonal arborizations of the neurons in the network. The parameters were optimized on the morphological shape characteristics of rat cortical L2/3 pyramidal neurons. Although all neurons in NETMORPH were generated with the same parameter values, all neurons were different from each other because of the stochastic nature of the neurite outgrowth rules in the model.

**Figure 3:**
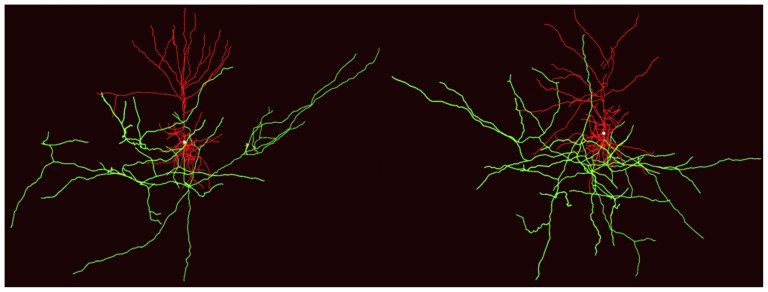
Examples of NETMORPH-generated L2/3 pyramidal neurons. Axons are shown in green, and dendrites are depicted in red. The neurons were grown with outgrowth parameters (Table 1) optimized on the dataset of L2/3 pyramidal cells from NeuroMorpho.org.

**Figure 4:**
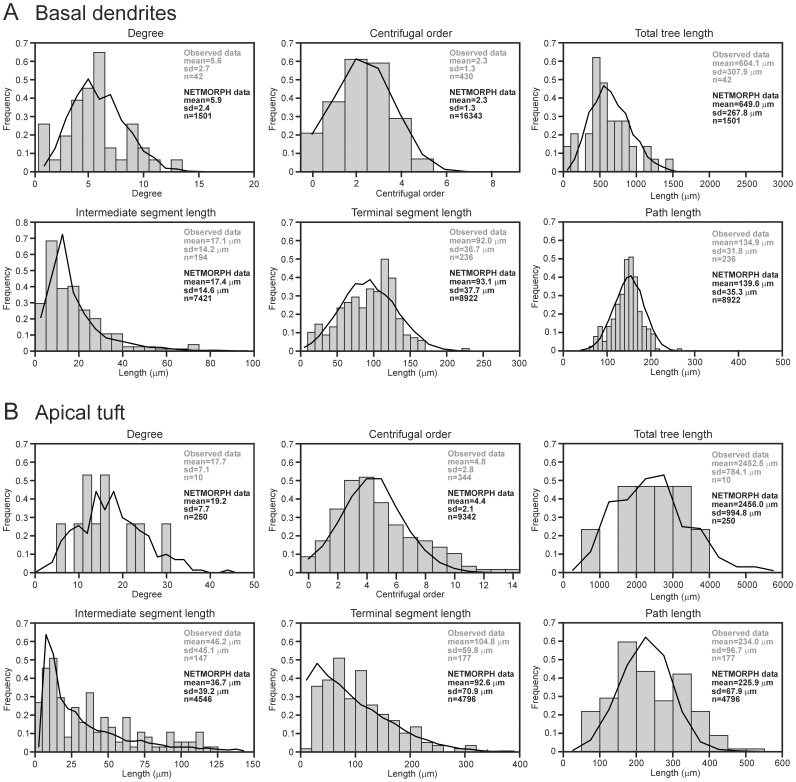
Distributions of dendritic shape characteristics of NETMORPH-generated and empirical L2/3 pyramidal cells. **A**, Basal dendrites. **B**, Apical tuft. The NETMORPH-generated neurons are shown by solid lines, and the L2/3 pyramidal neurons from the NeuroMorpho.org database are shown by grey bars. For the basal dendrites, the n-values of degree and total tree length refer to the total number of dendritic trees. For the apical tufts, the n-values of degree and total tree length refer to the total number of apical dendrites (equal to the number of neurons used). For both the basal dendrites and the apical tufts, the n-values of centrifugal order refer to the total number of segments (intermediate and terminal), the n-values of intermediate segment length refer to the total number of intermediate segments, and the n-values of terminal segment length and path length refer to the total number of terminal segments.

Figure 3 shows two instances of model-generated neurons. To demonstrate the similarity between the model-generated and empirical neurons, we compared the statistics of their morphological shape characteristics. Figure 4 shows the distributions, with means and standard deviations, of the various shape characteristics of the basal dendritic trees and apical tufts of the NETMORPH-generated neurons and empirical L2/3 pyramidal neurons. For each morphological shape characteristic, the basal dendrites and apical tufts of the model-generated trees have a good correspondence to those of the L2/3 neurons, with respect to both the overall form of the distribution and the mean and standard deviation of the distribution. Table 2 shows the means and standard deviations of the shape characteristics of the axonal trees of the NETMORPH-generated neurons. Again, these values compare quite well to those from the L2/3 pyramidal neurons. In conclusion, the parameter optimization procedure was successful in finding parameter values for creating L2/3-like pyramidal neurons in NETMORPH.

**Table 2:** Axonal shape characteristics of NETMORPH-generated neurons and L2/3 pyramidal neurons from the NeuroMorpho.org database.

	NeuroMorpho.org			NETMORPH		
Shape characteristic	Mean	Sd	n	Mean	Sd	n
Degree	47.5	15.3	4	46.8	29.5	250
Centrifugal order	7.42	3.62	376	7.24	3.21	23150
Total tree length (µm)	10870	4093	4	10496	7661	250
Path length (µm)	658	359	190	618	198	11700
Intermediate segment length (µm)	97.7	115	186	88.2	112	11450
Terminal segment length (µm)	133	119	190	138	163	11700

The n-values of degree and total tree length refer to the total number of axons (equal to the number of neurons used). The n-values of centrifugal order refer to the total number of segments (intermediate and terminal), the n-values of intermediate segment length refer to the total number of intermediate segments, and the n-values of terminal segment length and path length refer to the total number of terminal segments.

In the NETMORPH-generated network, synapses were formed where crossing axonal and dendritic line pieces came sufficiently close to each other (see Methods, Synapse formation). We subsequently characterized the emerging synapse distributions and network connectivity with a wide range of measures.

### Path distance of synapses to their post- and presynaptic somata

**Figure 5:**
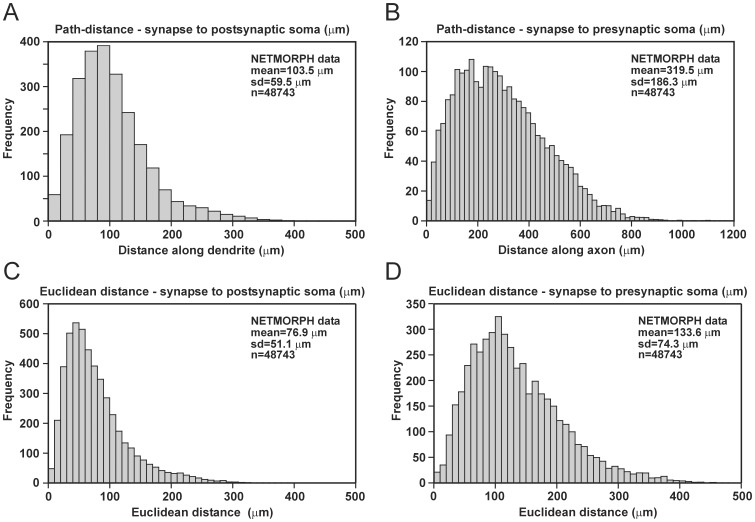
Distribution of path and Euclidean distances of synapses to their post- and presynaptic somata. **A**, Path distance to postsynaptic soma. **B**, Path distance to presynaptic soma. **C**, Euclidean distance to postsynaptic soma. **D**, Euclidean distance to presynaptic soma.

For each synapse in the generated network, we determined the path distance to its postsynaptic soma (distance along the postsynaptic dendritic tree) and to its presynaptic soma (distance along the presynaptic axonal tree). Figures 5A, B show the distributions of postsynaptic and presynaptic distances. Both types of distributions are clearly skewed, with an initial peak frequency followed by a tail of lower frequencies.

The distribution of postsynaptic distances (Fig. 5A) comprised synapses on both basal and apical dendrites. Synapses in the very tail of the distribution were mainly synapses on apical dendrites, which were longer than basal dendrites. The skewed shape of the distribution is partly determined by the amount of ‘dendritic mass’ around the soma and the distribution of synapses over the dendrites. As the path distance to the soma increases, the dendritic mass first rises, because of the increase in number of branches, and then falls off at longer distances, because of the termination of branches. The opportunities for synapse formation increase with increasing dendritic mass. Like our model findings, the experimental data of Le Bé et al. [47] also showed a skewed distribution of postsynaptic path distances on L2/3 pyramidal neurons. Although the synapses in the experimental study originated from layer 5 corticocallosal projecting neurons, their spatial positions on the dendrites (mn = 130 µm, sd = 133 µm, n = 55) compare quite well to our model outcomes (mn = 103.5 µm, sd = 59.5 µm, n = 48743).

The distribution of presynaptic distances (Fig. 5B) had a larger range than the distribution of postsynaptic distances. Axons were longer than dendrites, and synapses could therefore also have larger path lengths. Also here the amount of ‘axonal mass’ vs. path distance to the soma, which is determined by the axonal branching pattern, can partly explain the shape of the distribution. Compared with our model outcomes, the experimental data of Hill et al. [22] on synaptic connectivity between L5 pyramidal cells showed a similarly skewed distribution of presynaptic path distances, with exactly the same range of path distances and also a peak frequency around 200 µm.

### Euclidean distance of synapses to their post- and presynaptic somata

For each synapse in the generated network, we also determined the Euclidean distance to its post- and presynaptic somata. Both postsynaptic and presynaptic Euclidean distances were markedly shorter than the postsynaptic and presynaptic path distances, respectively (Fig. 5). Euclidean and path distances differed because of the turtuosity and changing orientation angles of the neurites in the axonal and dendritic arborizations. Like the distributions of path distances, the distributions of Euclidean distances were skewed, with an initial peak frequency followed by a tail of lower frequencies (Figs. 5C, D).

Synapses in the very tail of the distribution of postsynaptic Euclidean distances (Fig. 5C) were mainly synapses on apical dendrites. If synapses are uniformly spread over the dendritic membrane, the distribution indicates the amount of ‘dendritic mass’ as a function of Euclidean distance to soma. The shape of the distribution is influenced by the metrical and topological properties of the dendritic branching pattern, as well as the orientations and branching angles of the dendritic segments in 3D space. Compared with our model outcomes, the experimental data of Feldmeyer et al. [48] revealed a similarly skewed distribution of Euclidean postsynaptic distances on L2/3 pyramidal neurons. Although the synapses in this experimental study originated from L4 spiny neurons, their spatial positions on the dendrites (mn = 67.2 µm, sd = 33.6 µm, n = 59) compare quite well to our model outcomes (mn = 76.9 µm, sd = 51.1 µm, n = 48743).

The distribution of presynaptic Euclidean distances (Fig. 5D) shows that the range of distances was larger to the presynaptic soma than to the postsynaptic soma. Also here the amount of ‘axonal mass’ as a function of Euclidean distance to the presynaptic soma partly accounts for the shape of the distribution. Experimental data was not available for comparison.

Taken together, our results show that independently outgrowing neurons and simple proximity-based synapse formation rules yield pre- and postsynaptic distributions of synapse locations that are comparable to the available experimental data.

### Number of synapses per connection (connection strength)

**Figure 6:**
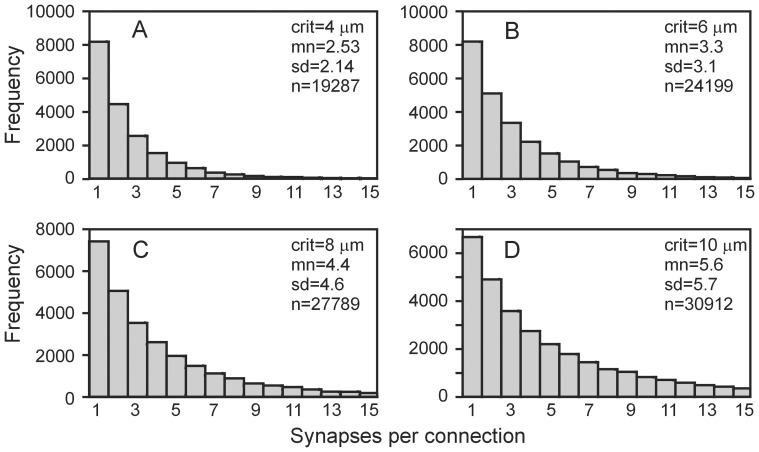
Frequency distributions of number of synapses per connection (connection strength) for different threshold distances for the formation of synapses. **A**, 4 µm. **B,** 6 µm. **C,** 8 µm. **D,** 10 µm.

Synapse locations in the generated network were determined on the basis of proximity between crossing axonal and dendritic line pieces (see Methods, Synapse formation). The larger the distance criterion, the more synapses will be created, and the larger the number of synapses per connection (connection strength). Figure 6 shows the frequency distributions of the number of synapses per connection in the generated network for distance criteria of 4, 6, 8 and 10 µm, which produced mean number of synapses per connection of 2.53 (sd = 2.14), 3.28 (sd = 3.1), 4.43 (sd = 4.6) and 5.57 (sd = 5.7), respectively. The experimental results of Feldmeyer et al. [48] on L2/3 pyramidal neurons showed a mean connection strength of 4.5 (sd = 0.5, n = 13), which falls within the range of our model outcomes. Figure 6 further shows that the frequency decreases with increasing connection strength, since the probability of having *n* synapses in a connection decreases with *n*.

We also compared our model outcomes with the semi-experimental results from Hellwig [20]. This author used 3D reconstructed rat visual cortex L2/3 pyramidal neurons that were placed at various distances from each other to determine synaptic connectivity as a function of cell distance. A synapse was considered possible when an axonal and dendritic branch shared a voxel with 1 µm side length. From the data in [20], we calculated that the mean numbers 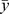 of synapses for a maximal cell separation of 93 µm (the radius of the 3D disc-shaped area in which the NETMORPH neurons resided) were 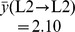, 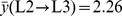, 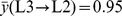 and 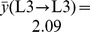. This gives an overall average of 1.85 synapses per connection among L2/3 cells. Assuming that a voxel overlap criterion of 1 µm corresponds to a 2 µm distance criterion (not shown in Fig. 6) for synapse formation in NETMORPH, we found a very similar value of 1.8 synapses per connection.

Taken together, our results show that the geometrical overlap between axonal and dendritic arborizations yields synapse numbers between neurons that are of the same order as the available experimental data.

### Connection probability vs. Euclidean distance between somata

**Figure 7:**
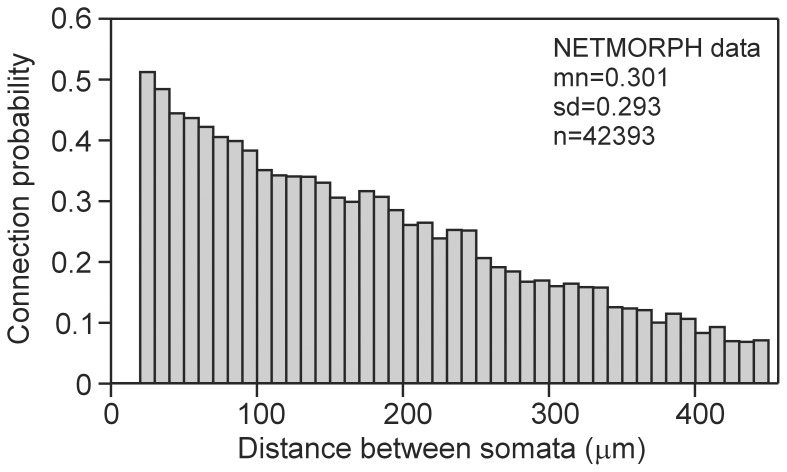
Connection probability between neurons as a function of Euclidean distance between their somata. Note that the minimum intersoma distance with which the network was created was 20 µm.

The connection probability between a pair of neurons at a given intersoma distance was estimated by dividing the number of connected neuron pairs (with at least one synaptic contact) at that intersoma distance by the total number of neuron pairs at that intersoma distance. Figure 7 shows that with increasing intersoma distance (30–440 µm), the connection probability decreased almost linearly from about 0.5 down to less than 0.1; the average connection probability was 0.301. This outcome is in good agreement with the semi-experimental data on L2/3 pyramidal neurons in rat visual cortex [20], which showed a gradually decreasing connection probability down to less than 0.1 at an intersoma distance of 500 µm.

Experimental data of Song et al. [49] on connectivity among thick tufted layer 5 pyramidal neurons in rat visual cortex, obtained with quadruple whole-cell recordings, showed a rather uniform connectivity probability of 0.116 up to an intersoma distance of 100 µm. This connection probability is somewhat lower than the probability in the NETMORPH-generated network for this distance range.

Using dual recordings, Holmgren et al. [50] estimated the connectivity among rat visual and somatosensory cortical L2/3 pyramidal neurons. They found that the pyramidal-pyramidal connection probability decreased from about 0.9 at short distance to about 0.2 at 140 µm. The latter value is somewhat lower than both our model outcomes and the Hellwig [20] results. The connection probability between pyramidal cells and fast-spiking interneurons was 0.5 for short distances.

To estimate the connectivity among thick-tufted layer 5 pyramidal neurons in rat somatosensory cortical slices, Perin et al. [51] recorded simultaneously from up to 12 cells, applying current pulses to each of these neurons and measuring the response in the other neurons. They found that the connection probability decreased almost linearly with intersoma distance from about 0.21 at 15 µm down to about 0.03 at 300 µm. Apart from an overall scale factor lowering the experimental values in Perin et al. [51] compared with our model outcomes, the NETMORPH results showed the same, almost linear, dependence of connection probability on intersoma distance.

In general, one might expect that in the experimental data the connection probabilities are somewhat lower than in our model. The electrophysiological experiments used functional synaptic connectivity to determine connection probabilities, whereas NETMORPH did this on the basis of candidate or potential synapses. In addition, Perin et al. [51] and Song et al. [49] considered L4 and L5 pyramidal cells, respectively, not L2/3 cells. Nevertheless, our results show that the geometry of axonal and dendritic arborizations together with proximity-based synapse formation already provides a good estimate of the connection probability between neurons.

### Frequency distribution of the Euclidean distance between connected somata

**Figure 8:**
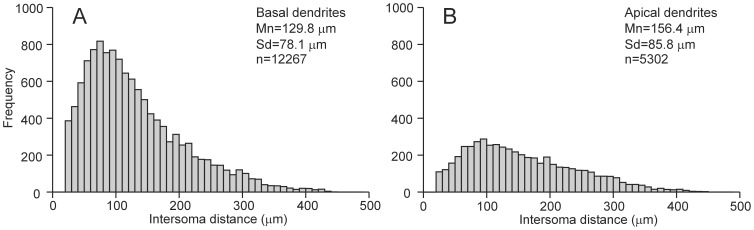
Frequency distribution of intersoma Euclidean distances between connected neurons. **A**, Connections via basal dendrites. **B**, Connections via apical dendrites. Note that the minimum intersoma distance with which the network was created was 20 µm.

In analyzing the distances between connected neurons, we made a distinction between connections via apical dendrites and connections via basal dendrites. Figure 8 shows the frequency distribution of intersoma distances between cell pairs in which the presynaptic cell had an axonal projection on the basal dendrites (Fig. 8A) or on the apical dendrite (Fig. 8B) of the postsynaptic cell.

For both types of connections, the frequency distribution of intersoma distances is clearly skewed, with an initial peak at about 100 µm and a tail of gradually decreasing frequencies at larger distances. Neurons can only become connected when their axonal and dendritic arborizations invade the same territory, i.e., if their axonal and dendritic density fields overlap. Apparently, at an intersoma distance of about 100 µm the axonal and dendritic arborizations had the highest joint densities, while becoming zero beyond about 500 µm. The mean of intersoma distances for connections via basal dendrites (mn = 129.8 µm, sd = 78.1 µm, n = 12267) was shorter than that for connections via apical dendrites (mn = 156.4 µm, sd = 85.8 µm, n = 5302), which can be explained by the greater spatial extension of the apical dendrite in comparison with that of a basal dendritic tree (see Fig. 4).

Experimental data on the synaptic connectivity between L2/3 supragranular pyramidal neurons in rat extrastriate visual cortex also showed a clearly skewed distribution of connection lengths [23], [52], but the peak frequency was at a higher connection length than in our results. However, the experimental data set [52] was incomplete, since only the longest branches of connections were reported.

### In- and out-degree and degree

We analyzed the degree of neurons on the basis of connections via either basal or apical dendrites. The in-degree of a neuron via its apical dendrite (basal dendrites) is the number of neurons from which it receives axonal projections onto its apical dendrite (basal dendrites). The in-degree distribution is built up from the in-degrees of all neurons in the network. The out-degree of a neuron via apical dendrites (basal dendrites) is the number of neurons to which it sends out axonal projections impinging onto apical dendrites (basal dendrites). Finally, the degree of a neuron, again separate for apical dendrite and basal dendrites, is the total number of neurons connected to this neuron by both incoming and outgoing axonal projections.

**Figure 9:**
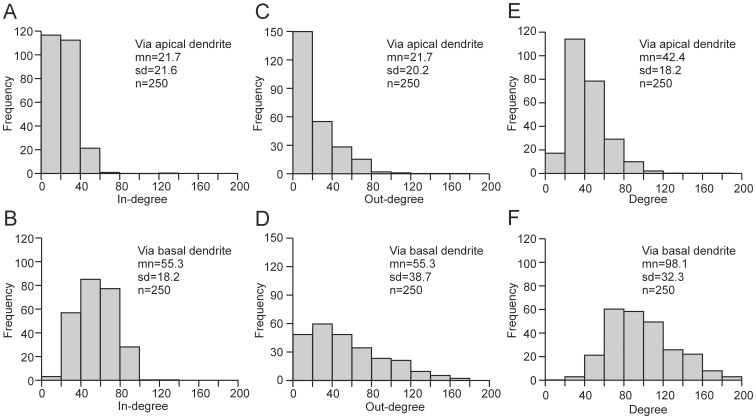
Frequency distributions of in-degree, out-degree and degree of neurons. **A**, In-degree for connections via apical dendrite. **B**, In-degree for connections via basal dendrite. **C**, Out-degree for connections via apical dendrite. **D**, Out-degree for connections via basal dendrite. **E**, Degree for connections via apical dendrite. **F**, Degree for connections via basal dendrite.

Figure 9 shows the various degree distributions. The in- and out-degrees via basal dendrites (Figs. 9B, D) were more than twice as large as the in- and out-degrees via apical dendrites (Figs. 9A, C). This can partly be explained by the greater size of the basal dendritic field in comparison with the apical dendritic tree. The average total length of individual basal dendritic trees was 649 µm (Fig. 4). The number of dendritic trees per neuron was on average 6, so the total length of the dendritic field of a neuron was on average 3894 µm. The mean total length of an apical dendrite was 2456 µm (Fig. 4). Thus, the basal field was 1.6 times as large as the apical dendrite. Furthermore, the apical dendritic field had its highest density further away from the soma than the basal dendritic field, which may also have resulted in a different overlap between axonal and basal dendritic field compared with that between axonal and apical dendritic field.

The degree distributions show a low frequency of highly connected neurons (hubs). For example, in the in-degree distribution via apical dendrites (Fig. 9A), there are a few neurons with 60–80 and 120–140 connections. The in- and out-degree distributions via apical dendrites show a monotonically decreasing frequency with degree (Figs. 9A, C), whereas the in- and out-degree distributions via basal dendrites (Figs. 9B, D) have a clear peak. Interestingly, the overall shape of the in- and out-degree distributions via apical dendrites (Figs. 9A, C) is different from the shape of the total degree distribution via apical dendrites (Fig. 9E), which implies that the set of neurons with a high in-degree was not the same as the set of neurons with a high out-degree.

A scale-free network requires a monotonically decreasing degree distribution that can be described by a power-law [53]. Since the total degree distributions in the model network were clearly peaked, there are no indications that the network was scale free. Experimental data on synaptic connectivity among rat somatosensory cortex cells [51] and monkey visual cortex cells [54] also revealed peaked degree distributions and therefore no evidence for scale-free connectivity.

### Small-world topology

Networks with a small-world connectivity structure are characterized by a mean shortest path length that is as small as in random networks and a clustering coefficient that is higher than in random networks (see Methods, Graph theoretical measures). We analyzed whether the connectivity in the NETMORPH-generated network had a small-world topology. In the NETMORPH-generated network and its randomized networks, all neurons were connected, so our analysis was not affected by the presence of sub-networks.

The NETMORPH-generated network had a mean shortest path length of 1.470 and a clustering coefficient of 0.622. Randomization yielded a mean shortest path of 1.541, so the original network had an even lower mean shortest path length than the randomized network (*λ* = *L*_orig_/*L*_rand_ = 0.954). The clustering coefficient in the original network was higher than that in the randomized network (*C*_rand_ = 0.460; *γ* = *C*_orig_/*C*_rand_ = 1.353), which is characteristic for networks with a small-world connectivity structure. The single coefficient proposed for measuring small-worldness, *γ*/*λ* [36], was therefore also higher than one (*γ*/*λ* = 1.418), indicating a small-world topology of the connectivity structure in the original network. Repeating the randomization runs for the small-world calculation led to the same results (two other runs: *L*_rand_ = 1.541, *C*_rand_ = 0.460, *γ*/*λ = *1.420; *L*_rand_ = 1.542, *C*_rand_ = 0.459, *γ*/*λ* = 1.420). Thus, independently outgrowing neurons and proximity-based synapse formation can already give rise to small-world connectivity.

Perin et al. [51] found that the connectivity structure among pyramidal cells in rat somatosensory cortex had a small-world topology, with a mean shortest path length of less than 2, similar to what we found in the NETMORPH-generated network. Gerhard et al. [54] obtained an average small-world coefficient *γ*/*λ* of 1.71 for the connectivity structure among cells in monkey visual cortex, which is slightly higher than that in our model network. However, Gerhard et al. [54] derived the small-world coefficient from multi-electrode recordings of electrical activity, and they showed that, since any electrophysiological recording constitutes a sub-sample of the network activity, this overestimated the true strength of the small-world structure of the network.

## Discussion

Local cortical circuits are composed of neurons with highly branched axons and dendrites that form a complex 3D meshwork of intertwining axonal and dendritic arbors [55]. In this intricate web of arbors, neurons can establish synaptic connections when their axonal and dendritic branches come within close proximity of each other [12]. Synaptic connectivity thus strongly depends on how the innervation of 3D space by axons and dendrites gives rise to locations where axonal and dendritic branches come within a threshold distance of each other. Whether outgrowing neurons thereby influence (by secreting chemical guidance cues) each other's axonal and dendritic trajectories and thus synapse locations, or whether chance encounters of independently outgrowing axons and dendrites can already account for synaptic connectivity, remains an issue of debate [19], [22].

To address this question, we generated, using our simulation framework NETMORPH [24], a network of independently outgrowing cortical L2/3 neurons in the absence of any extracellular guidance cues steering axons and dendrites. We subsequently explored what synapse distributions and synaptic connectivity patterns emerged from realistic neuronal morphologies in combination with synapse formation based solely on the proximity between axonal and dendritic branches. Synapse locations were thus fully determined by the 3D geometries of axonal and dendritic arborizations.

Previous studies examining the relationship between neuronal morphology and synaptic connectivity focused on particular aspects of connectivity, such as the extent to which the geometrical overlap between axons and dendrites can account for the number of synapses between neurons [18], [19], the number of synaptic connections as a function of intersoma distance [20], [21], and the spatial distribution of synapses on dendrites [22]. Kaiser et al. [23] investigated other features of connectivity, but used extremely simplified neuronal morphologies. They modeled axons as single straight lines and approximated dendritic trees by circles. In our study, we used realistic axonal and dendritic morphologies and analyzed many aspects of the emergent spatial synapse distributions and synaptic connectivity patterns. In the paper that introduced NETMORPH [24], only a few preliminary pilot findings were described aimed at showing the potential of NETMORPH for studying synaptic connectivity. The current study is the first full study in which NETMORPH is applied.

In our model-generated network of L2/3 pyramidal neurons, we found (i) skewed distributions of the postsynaptic locations (position on dendrites) and presynaptic locations (position on axon) of synapses; (ii) a connection probability between neurons that monotonically and almost linearly decreased with intersoma distance; (iii) a skewed distribution of connection lengths (distance between connected neurons); (iv) degree distributions (number of neurons with which a neuron is connected) that revealed hubs but no scale-free connectivity; and (v) a small-world topology of the synaptic connectivity structure.

In general, experimental data on synaptic connectivity within cortical networks are scarce because of the technical challenges involved in measuring large numbers of neurons. Nonetheless, we could compare our model outcomes with a number of suitable data sets from the literature. These data sets were not always the best imaginable but were the best available to us. The comparisons showed not only qualitative but in many cases also quantitative agreement between model outcomes and experimental data.

Both the shapes and the means of the distributions of postsynaptic locations of synapses agreed well with the experimental data, both in terms of path distance along dendrites to soma [47] and in terms of Euclidean distance to soma [48]. Likewise, the shape, range and peak value of the distribution of presynaptic locations of synapses, in terms of path distance along axon to soma, were comparable to those of experimentally observed distributions [22]. The number of synapses between connected neurons (connection strength) also fell well within the range of experimental values for cortical pyramidal cells [48].

The values of the connection probability between pyramidal neurons in the model-generated network were in line with the values reported in the experimental literature [49]–[51], especially when taking into account that the latter were mostly derived from electrophysiological recordings rather than from direct anatomical observations. In addition, both our model findings and the experimental data of Perin et al. [51] revealed an almost linear dependence of connection probability on intersoma distance.

The connection length distributions in the model-generated network and in the experimental data of Lohmann and Rorig [52] had a similar skewed shape. In fact, in a wide range of neural systems the connection lengths distributions have similar shapes, with an initial large peak and a flat tail representing longer-distance connections [23].

Like our model outcomes, the experimental data on synaptic connectivity among cortical cells [51], [54] revealed peaked degree distributions, the presence of hubs in the degree distributions, but no scale-free connectivity. Also as in the model-generated network, the connectivity structure among cortical cells exhibited small-world topology [51], [54].

Taken together, our model results indicate that realistic neuronal morphologies, simple geometry-based synapse formation rules and independently developing neurons are capable of producing networks with realistic synapse distributions, connectivity patterns and small-world properties. Given the many factors involved in the development of connectivity and the limited number of assumptions in the model, the qualitative and often also quantitative agreement between model outcomes and experimental data is surprising and not trivial. In the model, connectivity is determined on the basis of geometrical considerations and in the absence of extracellular guidance cues, and thus solely depends on the morphology of axons and dendrites and the spatial distribution of the cells. Connectivity is defined in terms of candidate synapses; whether functional synapses actually develop at locations of candidate synapses is an issue that is not considered in the model.

The outgrowth of a neuron's axon and dendrites was not influenced by the axons and dendrites of the other neurons in the network. Importantly, the parameter values of the neurite outgrowth model were optimized only for generating realistic neuronal morphologies, not for producing particular connectivity patterns. Synaptic connectivity was thus entirely an emergent property of the neuronal morphologies and the spatial distribution of the cells. The neurite outgrowth model in NETMORPH is a phenomenological model, in which all the growth actions are described as stochastic events. Such a stochastic description is appropriate when biological growth results from the concerted influences of many underlying mechanisms and interactions. Therefore, the model implicitly incorporates many factors affecting the development of neuronal morphology, including possible extracellular cues that may influence the shape of axons and dendrites. However, the external cues that are in this way implicitly implemented in the outgrowth model are non-specific, in the sense that in NETMORPH they have no role in steering axons and dendrites of any specific cell pair prior to synapse formation (see also [22]). Thus, the formation of synapses in NETMORPH was not, explicitly or implicitly, guided by signalling mechanisms such as extracellular chemical attraction and repulsion. In the nervous system, guidance cues may play an important role in the fine-tuning of synaptic connectivity and especially in the establishment of long-distance connections and the formation of large-scale connectivity patterns, but these were not considered in the present study.

Neurons in NETMORPH are generated on the basis of principles from neural development. The neurite outgrowth model implemented in NETMORPH uses stochastic phenomenological rules for growth-cone mediated neurite elongation and branching. The stochasticity gives rise to characteristic morphological variability between the generated neurons (Fig. 4). Thus, in our study, as opposed to other studies [20], [22], synaptic connectivity was not based on a single or limited set of exemplars of neuronal morphologies. As in real cortical networks, each L2/3 pyramidal neuron in the NETMORPH-generated network had a different detailed morphology but obeyed the statistical regularities characteristic of that neuron type.

The parameters of the neurite outgrowth model were optimized on the basis of morphological properties of experimentally reconstructed L2/3 pyramidal neurons made available on the NeuroMorpho.org database. Most of these reconstructions were obtained from sliced tissue. This means that parts of the dendritic and axonal arborizations that were outside the thickness of the slice were lost. We therefore took care to select only the most complete reconstructions from the database, i.e., neurons of which the cell body was in the center of the slice and the apical dendritic main stem was fully contained within the slice.

Our results suggest that accidentally overlapping branches from axonal and dendritic morphologies may to a large extent explain local synaptic connectivity. Dendritic morphology is, however, not fixed but can undergo significant alterations, for example in pathological conditions such as chronic stress [56]–[59], Alzheimer's disease [60], [61] and disorders associated with mental retardation [62]. Chronic stress induces extensive regression of pyramidal apical dendrites [58]. In Alzheimer's disease, various aberrations in dendritic morphology have been observed, including a reduction in total dendritic length [60], [61] and changes in the pattern of dendritic arborization [63]. These anomalies in dendritic morphology could, via their effect on the organization of synaptic connectivity and dendritic synapse distributions, affect cortical information processing and ultimately contribute to impaired cognition.

Both the NETMORPH results and the experimental data of Perin et al. [51] show a major linear component in the dependence of connection probability on intersoma distance (Fig. 7). Apparently, the increasing number of branches in axonal and dendritic arborizations further away from the soma can compensate for the reduced connection probability with distance. This finding of an almost linear dependence on distance is also relevant for models of neuronal network activity, in which often faster than linearly decreasing functions, such as exponential functions, are used to define neuronal connectivity [64]–[67].

Although degree distributions are difficult to obtain experimentally, they have a large impact on the dynamics of electrical activity in neuronal networks [4]. Therefore, in model simulations of cortical activity, such as liquid state machine simulations of cortical computation [68], realistic in- and out-degree distributions are desired, and the degree distributions obtained in NETMORPH may provide a first approximation.

Small-world topology is mostly studied with regard to the connectivity structure between different brain areas or different cortical networks [38], [39], [69], [70]. Only very few studies have attempted to determine whether small-world topology exists within cortical networks, at the level of synaptic connections between neurons [51], [54], [71], [72]. In simulation studies, it has been demonstrated that small-world topology can evolve from certain optimality considerations [40], developmental time domains for network formation [73], and special synapse formation rules [23] or synaptic plasticity rules [74]. Our results show that no particular axonal or dendritic outgrowth rules are necessarily required to create small-world topology and that independently outgrowing neurons, realistic neuronal morphologies and proximity-based synapse formation suffice to produce neuronal networks with small-world synaptic connectivity. The higher connection probability between nearby neurons (Fig. 7) may give rise to the high clustering coefficient characteristic of small-world networks, while the sparse long-range connections between neurons may yield the shortcuts required for a small mean path length. Although the values of the small-world coefficient *γ*/*λ* were not very much higher than one, they were in general agreement with those based on effective connectivity derived from multi-electrode recordings in cortical networks [54], [71], [72]. Functionally, small-world synaptic connectivity may improve memory recall [42] and lead to faster and more reliable synchronization [75]. Loss of small world topology has been observed in schizophrenia [10], [76] and Alzheimer's disease [11].

In conclusion, our results lend support to the view that the foundation of synaptic connectivity in local cortical circuits may largely be formed by accidental appositions between axonal and dendritic branches of independently outgrowing neurons [18], [19], [22]. These general connectivity patterns laid down by overlapping axons and dendrites may then be further refined by more specific mechanisms.
